# A Study on the Psychological Structure and Mechanism of Shared Chinese Cultural Identity of University Students

**DOI:** 10.3389/fpsyg.2022.943989

**Published:** 2022-07-19

**Authors:** Dan Zhu, Xunyi Lin

**Affiliations:** ^1^College of Chemistry and Materials Science, Fujian Normal University, Fuzhou, China; ^2^College of Education, Fujian Normal University, Fuzhou, China

**Keywords:** the shared cultural identity, psychological structure, mechanism, an intermediary chain reaction, university students

## Abstract

In previous studies, as an important object of inheritance and promotion of Chinese culture in Chinese colleges and universities, the psychological change mechanism of college students in the process of receiving cultural identity education has not attracted widespread attention. This study focuses on ideological and political education in colleges and universities, and innovatively believes that in order to improve college students’ identification with Chinese culture, and make them consciously “internalize in mind and externalize in practice,” it is necessary to explore their psychological structure and mechanism of action in the process of accepting identity education and take measures to actively intervene. The study employs the method of questionnaire investigation, acquiring 1,119 effective samples from 30 universities in Fujian province. By establishing the theoretical framework of “cognitive identity—emotional identity—ideological identity—behavioral identity,” analyzing and examining the data, it is found out that: (1) cognitive identity affecting behavioral identity positively in a noticeable way; (2) by raising individual emotional identity, cognitive identity strengthening university students’ behavioral identity; (3) cognitive identity inspiring behavioral identity with benefits of ideological identity; (4) it being verified that there exists an intermediary chain reaction between emotional identity, ideological identity, cognitive identity, and behavioral identity. As a result, the followings are proposed: (1) further integrating the worthy Chinese traditional culture into university curriculum and developing students’ cognitive education of traditional culture; (2) exploring the aesthetic dimension of worthy Chinese traditional culture and deepening the students’ emotional identity of traditional culture; (3) demonstrating the essence of the worthy Chinese traditional culture and advancing the students’ ideological identity of traditional culture; and (4) intensifying the practice of the worthy Chinese traditional culture and motivating the students’ behavioral identity of traditional culture.

## Proposition of Problems

The Resolution of the Sixth Plenary Session of the 19th CPC Central Committee pointed out that Xi Jinping’s thought of socialism with Chinese characteristics in the new era is Marxism in contemporary China in the 21st Century. It is the quintessence of Chinese culture and Chinese spirit and has demonstrated the new leap of Sinicization of Marxism. Among them, Xi Jinping’s thoughts on socialism with Chinese characteristics in the new era are regarded as the essence of Chinese culture and Chinese spirit, which fully demonstrates the CPC’s consistent attitude toward excellent Chinese traditional culture. Xi Jinping’s thoughts on socialism with Chinese characteristics in the new era are deeply rooted in the fertile soil of excellent Chinese traditional culture. It is a major theoretical innovation combining Marxism with China’s reality and excellent Chinese traditional culture. It is also a concentrated embodiment of contemporary Chinese cultural self-confidence, which provides fundamental guidance and spiritual support for our specific work of implementing the project of inheriting and developing excellent Chinese traditional culture. Colleges and universities are the highland of ideological and cultural education, and inheriting and disseminating Chinese culture are one of the important responsibilities of colleges and universities. In the process of inheriting and carrying forward Chinese culture at colleges and universities, college students are a unique and most critical carrier. College students’ identification with Chinese culture and the cultivation of cultural self-confidence are not only related to their own political identity and even national identity, but also the new requirements of socialism with Chinese characteristics for colleges and universities to carry out Chinese cultural identity education. It is an important indicator to examine the effectiveness of ideological and political education at colleges and universities.

However, in order to improve college students’ recognition of Chinese culture and make them consciously “internalize them in the heart and externalize them in action,” it is necessary to explore and actively intervene their psychological structure and action mechanism in identity education. As an important carrier of excellent cultural inheritance and an important base of ideological and cultural innovation, colleges and universities should not only actively undertake the important mission of leading college students to improve their spiritual level and promoting college students to highly identify with Chinese culture, but also publicize and guide them from the positive side. At the same time, we should pay more attention to grasp the multiple characteristics of college students’ group psychology. The current study investigates a group of Chinese undergraduates’ perceptions of Chinese culture and they all are acutely aware of themselves “being Chinese,” who specify aspects of Chinese culture that they felt such awareness could be attributed to He and Xiao (2021). College students are active in thinking. Although they have received school education for a long time and have strong political sensitivity, they also “belong to youth in terms of age, but they are highly educated and have a strong sense of political autonomy.” They may welcome and embrace today’s diverse and complex social trends. They sometimes are unclear about or do not know the excellent and backward ideas in Chinese traditional culture and cannot distinguish between the positive and negative factors of foreign culture. If they can be actively cultivated with positive guidance, it will be more conducive to promote college students’ cognition and behavior identity (Yang and Jiang, 2021). The psychological characteristics of college students are also special. College students are not only recipients, but also active participants and important communicators in identity education. Research has shown that college students’ self-construction plays an important intermediary role in the effect of cultural identity on Subjective Well-Being (Liu et al., 2018). If colleges and universities can deliberately arouse the emotional resonance of college students in the process of carrying out cultural identity education, it may further spread and enlarge among college students, and the effect will increase significantly. College students also have obvious group characteristics, such as the nature of network communication and peer characteristics, mainly demonstrated as follows: In the new media era, online interaction has become an indispensable means for college students in communication. College students rely more and more on the Internet to perceive the external world and obtain information, so the impact of the Internet as a “tool” cannot be underestimated; college students have a relatively small and concentrated circle of friends. The closeness of peer groups can quickly promote and influence each other, and provide reliable human resources for mutual communication. In fact, the Chinese excellent traditional culture of university students in colleges and universities to carry forward and spread process is a dynamic process, in order to make the idea and cultural spirit of the Chinese culture accepted by college students cognition to the emotion from the surface to the concept of identity to practice hard, you must follow the progressive education concept, the psychological impact mechanism in the study of college students in the process, Thus put forward the most effective education methods.

Based on the above, this study intends to adopt empirical methods, take college students in Fujian Province as the research object and the Chinese cultural identity of college students as the research content with the focus on the psychological structure and action mechanism of college students in the process of receiving Chinese cultural identity education. It is hoped to provide theoretical and practical support for colleges and universities to carry out cultural identity education for college students, and provide reference and basis for local governments to cooperate with the state to implement the project of inheritance and development of excellent Chinese traditional culture. The limitations of this study are noted and should be addressed by future research. For example, the findings should be interpreted with caution, because the sample was limited to the students in a normal university in Fujian province in China. There is a need, therefore, to include the students in other types and levels of universities in other regions of China, in future studies.

## Literature Review and Research Hypothesis

### Research Based on “Cultural Identity” and “College Students’ Identity of Chinese Culture”

To clarify the identity of Chinese college students to Chinese culture, we must first understand the concept of cultural identity and related issues. Cultural identity has been mentioned in various fields with their own interpretation. Take American psychologist Eriksson and British cultural research expert Hall as examples. Eriksson believes that cultural identity refers to the positive recognition of the most meaningful things of the nation formed by the members of a group living together in the national community for a long time. Hall regards cultural identity as a dynamic and constantly constructing production process (Erikson, 1968). It is a cultural identity reached by people after the subjects form the same view of the world through the interaction between the subjects and other subjects. Most researchers at home and abroad mainly discuss the issue of cultural identity in the field of national identity and ethnical identity (Zou, 2014). Samuel, American political scientist Huntington put forward the view that culture reflects power. He believes that culture always uses its power to promote its values, practices, or systems to other societies, and emphasizes that cultural identity is the most meaningful thing for most people (Samuel, 1996); in China, culture has always been regarded as the blood of the nation and the spiritual home of the people. Cultural identity has played an important role in the formation of the national cultural psychology of Chinese integration and national unity, and in the stable development of our country and society. Chinese cultural identity not only affects people’s attitude toward the country and nation, but also affects people’s behavior toward the country and nation (Wang et al., 2017). Many researchers propose that cultural identity is the link between national identity and national identity (Meng, 2014); through empirical investigation, some studies have found that national identity has a positive predictive effect on the integration strategy of national culture and mainstream culture (Wang and Zhang, 2018). They proposed to strengthen national identity through cultural identity and promote national identity through common historical destiny and collective memory (Shen, 2015).

At present, the domestic research on college students’ Chinese cultural identity is also developing. College students are the most deserving and concerned group. Whether they identify with the culture of their own nation or not will even affect the mainstream status of the culture. Especially in contemporary China, as socialism with Chinese characteristics has entered a new era, the fine traditional Chinese culture has become more prominent as the ideological source and spiritual support of socialist core values, and should play an important role in the cultivation of talents in universities, especially in the ideological and political education of college students. College students’ true identification with the excellent traditional Chinese culture is bound to be the key to carry out ideological and political education in colleges and universities. Many researchers have carried out relevant theoretical research according to the characteristics of their discipline; some researchers conducted a sampling survey of many domestic colleges and universities to explore the current situation of college students’ recognition of excellent Chinese traditional culture and put forward countermeasures; some researchers also focused on ethnic identity and paid attention to the mainstream cultural identity of college students of ethnic minority; and other researchers have conducted empirical research on college students’ satisfaction and cultural identity in setting up school-based courses of Chinese excellent culture at colleges and universities. However, from the existing research situation, in recent years, although there are many researchers and the theories and methods adopted are constantly improving, especially in the empirical research, many useful explorations have been made, but most of them ignore an important problem: College students’ cultural identity is a gradual and dynamic process. Identity needs to be established step by step, not just as a state of existence. The individual psychological activities of college students are also changing. Therefore, the psychological structure and mechanism of college students in the process of identity deserve our attention, and the existing results are not enough to provide a direct reference for this study.

### Research Hypothesis

From the point of view of connotation, cultural identity can be regarded as social individual understands of culture and consensus identification, acceptance and finally achieve psychological adaptation approval. This research is mainly based on college students’ psychological reaction mechanism in the process of cultural identity to explore further, so we proposed from the perspective of psychology, the Chinese cultural identity in college ideological and political education work, and sufficient demonstration college students, the process of the identity of the Chinese culture to accept to better grasp the college students the necessity and importance of the Chinese cultural identity. This study puts forward the following hypotheses:

*H1*: cognitive identity has a positive impact on behavioral identity.

The premise of college students’ identity of Chinese culture is cognition. Without extensive and in-depth cognition, there will be no solid and profound identity. The degree of cognition determines the quality of identity (Shen, 2015). Cognition is the structure of cognition and understanding of objective things in an individual’s mind, that is, the mark formed by the repeated leadership of relevant contents and spiritual values in the process of personal growth. The cognitive identity of college students to Chinese culture is the initial identity of culture under the interpretation of memory object, and it is also the most prerequisite of cultural identity. Previous studies have shown that there is a significant positive correlation between college students’ cognitive identity of Chinese culture and their final behavioral identity. Some researchers point out that cognitive identity is the forerunner of the realization of mainstream ideological identity. Generally speaking, the deeper the understanding of the content of the mainstream ideology, the more conducive it is to form a firm emotional identity, belief identity, and will identity, and the easier it is to standardize its words and deeds and realize behavior identity according to this theoretical thought and value standard. Some studies have also proposed that the investigation of college students’ recognition of excellent Chinese traditional culture should be carried out from three aspects: knowledge recognition, concept recognition, and value recognition of excellent Chinese traditional culture, which defines the primary position of cognitive recognition (Cheng and Xiong, 2016). Other researchers have directly discussed the relationship between cognitive identity, behavioral identity, and cultural identity, and proposed to build a realistic path to enhance young students’ cultural cognitive identity and cultural self-confidence by expanding cultural cognition.

*H2*: emotional identity is an intermediary variable between cognitive identity and behavioral identity.

In the acceptance of college students’ cultural identity, in addition to cognitive identity, there are other intermediary elements that can directly affect college students’ behavioral identity. Emotional identity can strengthen and protect cognitive identity. The formation of emotional identity can be regarded as a positive emotional experience. In the formation and change of attitude, emotion can simplify the role of the whole identity process. Through various ways of information processing, emotion plays a driving and organizing role in cognition (Meng, 2005). The process of identification requires both cognitive reasonable acceptance and positive inner emotional experience. The two complement each other, and finally achieve a harmonious and unified state within the individual (Jiang, 2018). Nowadays, more and more people regard human cognition and emotion as two inseparable interactive parts, and observe how individual cognitive ability and emotional response are affected in more social situations (Jiang, 2018). Some studies have shown that different cognition will inevitably lead to different subjective emotional experiences, and different subjective emotional experiences will inevitably lead to different explicit behaviors (Jiang et al., 2019). At the same time, some studies have proposed that emotion has a great incentive effect on people’s directional behavior, and individual emotional identity is the internal driving force of their directional behavior (Wang, 2018). Emotional identity is not only the psychological foundation of cognitive identity, but also determines the sustainability and effectiveness of behavioral identity. In behavioral psychology, psychology and behavior are closely related, and there is a two-way interactive relationship between emotion and behavior (Jiang, 2018). Some scholars have demonstrated the influence of emotional identity on cognitive identity to behavioral identity in reverse, and put forward the problem that college students have low cognitive emotion toward Chinese culture. Some scholars mentioned in the research on the consciousness of the Chinese national community that “identification is not achieved overnight. Similar to demand, it is also a gradual increasing process from low level to high level, which is divided into three stages: cognitive identification, emotional identification, and behavioral identification. As the intermediate stage of identity, emotional identity is not only based on cognitive identity, but also affected by behavioral identity” (Lin et al., 2021).

*H3*: conceptual identity is an intermediary variable between cognitive identity and behavioral identity.

Empirical research shows that there is a positive correlation between cognitive identity and conceptual identity. Conceptual identity is a psychological choice based on people’s cognitive understanding of culture (Tang, 2010). Cognition here refers to the definite ideological will and spiritual belief formed based on rational cognition (Wang and Wei, 2018). “Social identity theory” provides theoretical support for the mutually promoting relationship between cognitive identity and conceptual identity. The theory puts forward that social identity is an interactive process in which social groups give the connotation of self to individuals and individuals belong to social groups. Previous studies have shown that people want to belong to groups that are approved, different from other groups, and can bring them positive comments. Through the comparison between groups, people will regard the group they belong to as psychologically unique. At the same time, when they are connected with relevant groups, people will more affirm their own group (Wang, 2009). There are also researchers combined with John Turner’s self-classification theory points out that the process of self-classification is a cognitive process. If cognition is comprehensive and systematic, the individual’s social identity to the group is easy to form and develop (Wang et al., 2017). In other words, when people classify themselves and form the characteristics of social identity such as group uniqueness, it shows that the degree of people’s cognitive identity is deepened, the cognition will become more rational, and can be promoted to the level of will and belief to form conceptual identity. Conceptual identity is the sublimation of cognitive identity, which promotes the transformation of students from “passive acceptance” to “active identity,” and provides a stable and long-term internal driving force for behavioral identity (Zhao and Feng, 2018). Mead, an American sociologist, clearly put forward the view that attitude is the beginning of behavior for the first time, and believed that people enter a continuous behavioral process chain from the beginning of reaction (Ding et al., 2009). Only when culture is recognized, understood, accepted, recognized, and internalized can it have real identity. This recognized culture can produce a strong sense of action (Tang, 2010). Conceptual identity is not only an important driving force from cognitive identity to behavioral identity, but also an important indicator to affect and measure college students’ acceptance of Chinese culture.

*H4*: cognitive identity affects behavioral identity through the chain intermediary of emotional identity and conceptual identity.

The formation of identity requires both cognitive rational choice and emotional attribution. Finally, the practice of behavior constitutes the whole process of identity (Jiang, 2018). Previous studies have indicated that cultural identity is human’s tendentious consensus and recognition of culture. This consensus and recognition is the sublimation of human cognition of nature, and forms the thinking criteria and value orientation that dominate human behavior (Zheng, 1992). Cultural identity can be understood as the consensus recognition, acceptance, and approval of the subject based on the cognition and understanding of culture and its systematic nature, which is expressed through specific behaviors in daily life (Jian, 2020). In the process of forming college students’ cultural identity, “everyone must first enter this culture and must learn and absorb culture,” which shows the foundation and primacy of cognitive identity in this process; there is also a significant positive correlation between emotional identity and conceptual identity. Rational identity is the further sublimation of emotional identity, a deeper emotion, and a sound social mentality. It shows that “an individual realizes that he belongs to a specific nation through the process of self-classification, and the national identity he obtains will give him a certain emotional and value significance. The emotional significance is largely determined by blood relationship, while the value significance is more determined by national culture” (Guo et al., 2017). This is also the psychological resonance of an individual’s emotional love for a specific cultural connotation, so as to bring it into the self-concept and behavior world, resulting in value and significance. Rational behavior theory also shows that people are rational. Before making a certain behavior, they will synthesize all kinds of information to consider the significance and consequences of their own behavior. Therefore, some studies have proposed that identity comes from thought and falls into action. Behavioral identity is the externalization stage of cognition and emotion. The subject will put it into action, either based on identity consciousness, or based on value consideration, or based on the burst of emotion (He et al., 2020).

## Data Source and Research Design

### Data Source

The target population of this study is college students (including postgraduate students and doctoral students) from 13 colleges and universities in Fujian Province. In November 2021, 1,300 questionnaires were distributed through the Questionnaire Star software, and 1,119 valid questionnaires were recovered, with an effective recovery rate of 86.1%. On the whole, the survey objects consider the gender ratio, professional coverage, grade, educational level, nationality, belief, family education, and other aspects, which are well representative. Please see the Table 1.

**Table 1:** Frequency analysis.

Variable	Options	Frequency	Percentage (%)	Cumulative percentage (%)
Gender	Male	369	32.98	32.98
	Female	750	67.02	100.00
Nationality	Han Chinese	1,057	94.46	94.46
	Minority	62	5.54	100.00
Originality	Urbanization	467	41.73	41.73
	Village	652	58.27	100.00
Student Identity	Freshman	452	40.39	40.39
	Sophomore	329	29.40	69.79
	Junior	174	15.55	85.34
	Senior	36	3.22	88.56
	Master	124	11.08	99.64
	PhD	4	0.36	100.00
Major	Literature	205	18.32	18.32
	Science	236	21.09	39.41
	Engineering	198	17.69	57.10
	Medical	90	8.04	65.15
	Art	10	0.89	66.04
	Agronomy	7	0.63	66.67
	Others	373	33.33	100.00
Political Status	Party member	106	9.47	9.47
	The communist youth league	777	69.44	78.91
	Masses	236	21.09	100.00
Religion	The communist youth league	1,047	93.57	93.57
	Be religious	72	6.43	100.00
Total		1,119	100.0	100.0

### Research Design

#### Questionnaire Compilation

On the basis of combing and learning from the existing literature research, this study investigated college students of different majors in different colleges through interviews, invited experts in relevant research fields to read and approve the dimension indicators and item contents of the research, and compiled the Pre-questionnaire on College Students’ Cultural Identity. The questionnaire is scored with Likert five-scale. 1 means completely disagree, 2 means slightly disagree, 3 means uncertain, 4 means slightly agree, and 5 means fully agree. In this study, 150 undergraduates from Fujian Normal University were selected for a pre-questionnaire survey. After the test and correlation analysis of the data, based on the respondents’ feedback, the content expression of the questionnaire was improved and the items were deleted, and the unclear and ambiguous parts were revised after the final evaluation and test by peer experts, so as to ensure the accuracy of the written statement and the applicability of the cultural situation. Finally, the questionnaire is divided into two parts: (1) Basic information of respondents; (2) There are four subscales: cognitive identity, emotional identity, conceptual identity, and behavioral identity, with a total of 34 items.

#### Reliability and Validity Test

In this paper, the reliability test and exploratory factor analysis (EFA) of the self-made scale were carried out. According to the analysis results of SPSS, after deleting the items of the four dimensions respectively, the KMO value of the scale is 0.975, and through the spherical test (*p* < 0.000, the cumulative variance contribution rate is 81.212%), indicating that the scale is suitable for further EFA. According to the set structure, the principal component analysis method is adopted. By setting a fixed number of factors to be extracted, the rotation axis method is the maximum variance method of the orthogonal rotation axis method, and the minimum coefficient of 0.5 is prohibited. Among them, there are 17 measurement items of cognitive identity, 12 measurement items of emotional identity, 14 measurement items of conceptual identity, and 11 measurement items of conceptual identity. After EFA, delete the items with factor load less than 0.5. Finally, each observation item can be well distributed on its own factor load, and get four common factors: cognitive identity, emotional identity, concept identity, and behavior identity, which meet the research expectation. Among them, cognitive identity includes 10 measurement items, emotional identity includes six measurement items, conceptual identity includes 10 measurement items, and behavioral identity includes eight measurement items. The specific data are shown in Tables 2, 3.

**Table 2:** KMO and Bartlett’s test.

Kaiser-Meyer-Olkin measure of sampling adequacy		0.975
Bartlett’s Test of Sphericity	Approx. Chi-Square	52749.065
	*df*	561
	Sig.	0.000

**Table 3:** Rotated component matrix.

	Component			
	Conceptual identity	Cognitive identity	Behavioral identity	Emotional identity
LN6	0.864			
LN5	0.863			
LN8	0.848			
LN14	0.826			
LN13	0.816			
LN9	0.787			
LN3	0.779			
LN2	0.777			
LN12	0.723			
LN10	0.706			
RZ5		0.834		
RZ6		0.831		
RZ1		0.830		
RZ12		0.797		
RZ17		0.767		
RZ2		0.755		
RZ1		0.717		
RZ8		0.712		
RZ3		0.706		
RZ16		0.665		
XW9			0.749	
XW8			0.729	
XW5			0.717	
XW6			0.709	
XW10			0.697	
XW7			0.697	
XW4			0.670	
XW1			0.621	
QG5				0.737
QG6				0.703
QG3				0.691
QG1				0.651
QG4				0.646
QG8				0.595

Extraction Method: principal component analysis. Rotation Method: varimax with Kaiser Normalization.

Cronbach’s of the scale population *α* coefficient is 0.981, Cronbach’s of cognitive identity scale *α* coefficient is 0.951, Cronbach’s of emotional identity scale *α* coefficient is 0.967, Cronbach’s *α* conceptual coefficient is 0.980, and Cronbach’s of behavioral identity scale *α* coefficient is 0.970. As shown in Table 2, the questionnaire has good reliability (Table 4).

**Table 4:** Reliability test.

Scale	Cronbach’s *α* coefficient	Number of items
Total	0.981	34
Cognitive identity	0.951	10
Emotional identity	0.967	6
Conceptual identity	0.980	10
Behavioral identity	0.970	8

**Figure 1:**
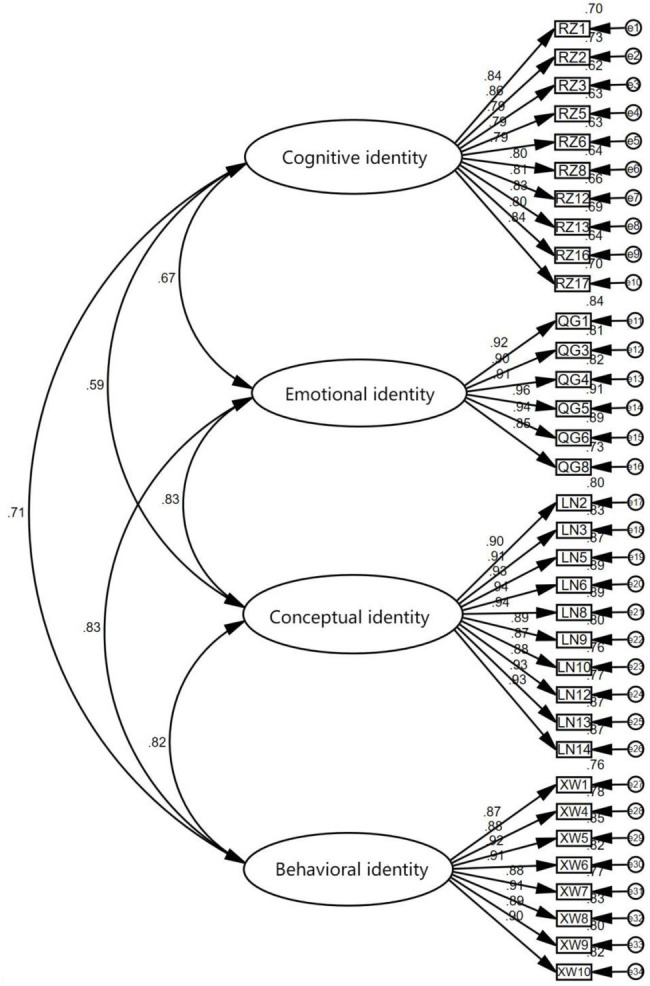
Confirmatory factor model.

In this paper, Amos24.0 confirmatory factor analysis (CFA) was performed on the results of EFA to further test the effectiveness and stability of the factor structure, and the combined reliability was used to measure the reliability quality of each dimension. The average variance extraction amount AVE is used to explain how much of the variance of the dimension comes from the measurement error. The larger the measurement error is, the smaller the value of AVE is, and the ideal standard is 0.5. The standardized factor load coefficients of each item in the sample data of this study are greater than 0.8, and the critical ratios are higher than 1.96, which are significant and statistically significant. The combination reliability of each factor is higher than 0.9, which has good combination reliability, and the average variance extraction value AVE of the factor is higher than 0.6, so the aggregation validity of the model is good. This study adopts the maximum likelihood estimation method to estimate the scale model. The main fitting indexes of the model are: RMSEA is 0.095, less than 0.1, GFI = 0.736, NFI = 0.891, CFI = 0.900, IFI = 0.900. The overall fitting of the model is acceptable, and the CFA model is shown in Figure 1.

## Data Analysis Result

The four measurement dimensions: cognition, emotion, concept, and behavior identity are standardized. According to the model in the process plug-in of SPSS macro program proposed by Hayes (2013), this paper examines the intermediary role of emotion and idea identity between cognition and behavior identity.

**Figure 2:**
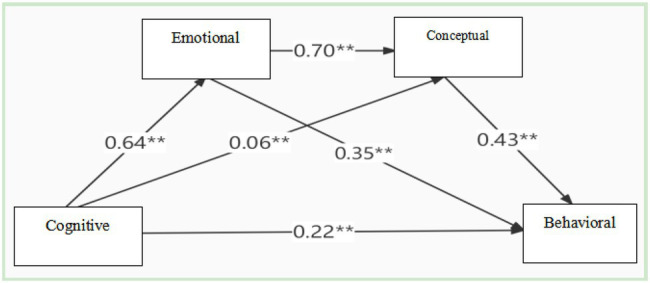
The bootstrap method of deviation correction is used to test the intermediary effect to check whether the intermediary effect is significant. Reset the sampling setting to 2,000 times, and calculate the 95% deviation correction CI. If the 95% confidence interval (95% CI) does not include 0, it indicates that the intermediary effect is significant. The results show that emotional identity and conceptual identity play multiple mediating roles in the relationship between cognitive identity and behavioral identity. **p < 0.01.

First of all, take cognitive identity as the independent variable and behavioral identity as the dependent variable to construct model 1 to explore the direct effect of cognitive identity on behavior; secondly, add emotional identity and conceptual identity as independent mediating variables, build model 2, and explore their parallel mediating effects; and finally, on the basis of model 2, establish the link between emotional identity and conceptual identity, that is, the chain intermediary path of “cognitive identity → emotional identity → conceptual identity → behavioral identity,” build model 3, and explore the chain intermediary effect of the two. The model diagram is shown in Figure 2.

According to the test results of mediating effect presented in Table 3 and Figure 2, emotion and idea play a partial mediating role in the relationship between cognition and behavior, and the mediating effect is significant. The mediating effect value is 0.44, 95% CI is [0.38, 0.51], accounting for 66.67% of the total effect of cognition on behavior. The total mediating effect consists of three indirect effects: the first path is cognition → emotion → behavior. The effect value is 0.22 and 95% CI is [0.16, 0.29], accounting for 33.33% of the total effect. The second path is cognition →concept → behavior. The effect value is 0.03 and 95% CI is [0.01, 0.05], accounting for 4.55% of the total effect. The third path is cognition → emotion → concept → behavior. The effect value is 0.19 and 95% CI is [0.15024], accounting for 28.79% of the total effect. The CI score of all mediating effects does not include 0, indicating that all mediating paths are established, and the mediating effects of cognitive identity on behavioral identity through emotional identity, conceptual identity, and emotional identity → conceptual identity mediating chain are significant. The direct effect value from cognitive identity to behavioral identity is 0 22, the total effect value is 0.66 (Table 5).

**Table 5:** Intermediary effect analysis.

	Effect	BootSE	95% CI	Effect amount
Direct effect	0.22	0.020	[0.18，0.25]	33.33%
Cognition → emotion → behavior	0.22	0.03	[0.16，0.29]	33.33%
Cognition → concept → behavior	0.03	0.01	[0.01，0.05]	4.55%
Cognition → emotion → concept → behavior	0.19	0.22	[0.15，0.24]	28.79%
Total intermediary effect	0.44	0.03	[0.38，0.51]	66.67%
Total effect	0.66	0.02	[0.62，0.70]	

## Conclusion

### Analysis and Discussion

#### The Direct Effect of Cognitive Identity on Behavioral Identity

Firstly, this study found that cognitive identity significantly positively predicted behavioral identity, which was in line with hypothesis 1. It can be seen from the existing data that the direct effect of cognitive identity on behavioral identity accounts for 33.33% of the total effect, and the direct effect value is 0.22. In other words, with the improvement of college students’ cognitive identity, their behavioral identity also improves accordingly; On the contrary, if college students’ cognitive identity is not high, they show a low level of behavioral identity. Previous studies have similar theoretical explanations, “From the personal perspective, ideological identity is a process in which individuals recognize and accept the ideas, concepts and value norms of a certain ideology, integrate them into their own ideas and value system, form an organic part of their own ideology, and then dominate and control their own behavior to make it meet the requirements of the ideology” (Nie, 2010). The foothold of cultural identity is behavioral identity, in which the problem of cognitive identity, interpretation knowledge, emotion and idea identity, interpretation letter, and finally point to the problem of explaining practice through behavioral identity.

Cognition, emotion, idea, and behavior are the four dimensions of college students’ psychological response construction of Chinese cultural identity, which are interrelated and interact with each other to form a closed loop. Cognitive identity is the primary stage of cultural identity, which is the habit, ability, and thinking acquired by individuals under the influence of the objective world, and the rational guidance for college students to learn and absorb Chinese culture. Emotional identity is the driving layer of cultural identity. It is a kind of approval and affirmative attitude generated on the basis of people having a deep and comprehensive understanding of objective things. It is a kind of incentive and promotion for college students to rise from rational cognition to emotional investment. Concept identity is the core level of cultural identity. It is a common cultural concept, ideal, and belief formed in the process of identity. It is the sense of pride and belonging of college students to Chinese culture in their hearts and beliefs. Behavioral identity is the highest level of cultural identity, which is not only the last link of identity structure, but also the highest goal of cultural identity. To test the achievements of cultural identity, it is to see whether college students have really achieved behavioral identity.

#### The Intermediary Role of Emotional Identity and Conceptual Identity

This study also further reveals the indirect path of how cognitive identity affects college students’ behavioral identity, that is, it further discusses the intermediary role of emotional identity and conceptual identity between cognitive identity and behavioral identity.

Firstly, emotional identity plays an independent intermediary role between cognitive identity and cultural identity, that is, cognitive identity can enhance individual emotional identity and then enhance college students’ behavioral identity. Hypothesis 2 is verified. And the effect amount of this path accounts for the largest proportion in the total indirect effect, the effect value is 0.22, and the intermediary effect accounts for 33.33% of the total effect. The intermediary effect is significant, which is the most important intermediary variable. The conclusion of previous research has been verified, “in the process of college students forming their awareness of identity with the Chinese nation, emotion also have the functions of awakening, stimulating, mediating, regulating, and strengthening. Emotional identity is the key for college students to confirm the common identity of the Chinese nation and form an identity consciousness for the Chinese nation” (Sun, 2021). It can be considered that emotion plays an important role in the psychological structure of cultural identity, guides and promotes the formation of behavior, and provides the initial composition for the formation of identity. “Emotional identity strengthens and protects cognitive identity” (Jiang, 2018). It can be said that emotional identity is the intermediate level connecting individuals in cultural cognition and behavioral identity, which constructs the identity form of emotional experience and marks the completion of the internalization process of identity. Therefore, emotional identity is an important part of the transition from cognitive identity to behavior, and it is the preparation stage to realize behavioral identity.

Secondly, conceptual identity plays an independent intermediary role between cognitive identity and behavioral identity, that is, cognitive identity can affect college students’ behavioral identity through conceptual identity. Hypothesis 3 is verified. The effect amount of this path accounts for a relatively small proportion of the total indirect effect, and the effect value is 0.03, which is significant. This shows that college students’ cultural identity is the acceptance and recognition of ideas, which can have a positive aftereffect on behavioral identity, that is, to enhance the further turn of individuals from cognition to behavior. This positive aftereffect will also directly promote the psychological identity logic of college students to Chinese culture and further improve the level of college students’ behavioral identity. This path accounts for a relatively small proportion of the total indirect effects. The reason may be that the impact from cognition to behavior may be more to arouse positive energy through emotion, and then rise to the understanding of ideas. Emotional identity and rational identity are the common collaborative influencing factors between cognitive and behavioral identity. Cognitive identity is the basis of the transformation of behavioral identity. “Emotional identity, as the connecting point of the transformation from value cognition to value identity, and as the starting point of the process of value cognition, constitutes the basis and premise of concept identity and behavior identity” (Wang et al., 2018). Emotion and idea are the executive power of behavior change. Only after obtaining the knowledge cognition about Chinese culture and positive thinking about knowledge can we have a strong positive cognition, trigger emotional resonance, rise to the psychological acceptance of ideas, and finally take positive action to improve personal cultural identity experience. “As a comprehensive sublimation of rational cognition and emotional experience, conceptual identity abandons the one sidedness of rational cognition and the transience of emotional identity, and provides a stable and long-term internal driving force for the practice of value” (Wang et al., 2018).

#### The Chain Intermediary Role of Emotional Identity and Conceptual Identity in the Influence of Cognition on Behavioral Identity

Finally, this study also reveals that emotional identity and conceptual identity have a chain intermediary effect between cognitive identity and behavioral identity. Hypothesis 4, the core theoretical presupposition of this study, has been verified. The chain intermediary effect accounts for 28.79% of the total effect. The level of intermediary effect is significant, that is, college students’ cognitive identity can affect individual behavior identity through the chain intermediary effect of emotional identity and conceptual identity. This also provides a possible explanation for how cultural cognitive identity affects college students’ overall cultural behavior identity. “Ideological identity begins with cognitive identity and ends with behavioral identity. It basically follows the step-by-step development process from cognitive identity, emotional identity, and internalized identity to practical identity. The emergence of the latter level of identity does not come out of thin air, but comes from the development of the previous stage of identity, which can be regarded as the basic law of the development of ideological identity” (Wang et al., 2018).

The results show that emotional identity is an important part of cognitive identity acting on behavioral identity through chain mediation. Cultural cognitive identity is a psychological guide for individuals to accept knowledge, process information, and judge value in the process of socialization. It expresses the psychological mechanism that individuals pay attention to, understand, experience, and accept specific cultural forms, and finally recognize. In this process, the positive experience of emotion is the recognition result of cultural learning and acceptance. This emotional experience can further deepen the transformation from cognition to behavior recognition and strengthen recognition. Conceptual identity can not only play an independent role between cognitive identity and cultural identity, but also affect behavioral identity under the role of emotional identity. Specifically, conceptual identity is to further deepen the effect of identity on the basis of emotional identity. Its driving force comes from the rational judgment of individual cultural understanding in the context of socialization. Conceptual identity will further externalize the individual’s understanding, and acceptance of a specific culture, so as to establish their own value attitude, which is self-conscious and lasting. Therefore, if college students have a high degree of cognitive identity to culture, they are more likely to further form concept identity under the action of emotional identity, so as to strengthen behavior identity and lead practice. It can be said that the practical level of cultural identity is expressed as behavioral identity, which belongs to the highest level of cultural identity. Its basic driving force comes from the individual’s emotions and ideas in cultural identity. The basic process is to constantly accept the value form of a specific culture in the individual’s cognitive activities of culture, and embody the respect for a certain cultural form in behavior.

### Enlightenment and Suggestions

Chinese culture is the crystallization of 5,000 years of wisdom of the Chinese nation. It is the foundation for China’s development and inheritance. Colleges and universities have always been regarded as the front line of cultivating and practicing socialist core values, and cultural education has a great mission. Turning the “root” and “soul” of Chinese culture into important spiritual wealth related to the growth and success of college students is not only the realistic demand of realizing the fundamental task of moral cultivation in colleges and universities, but also the due meaning of the reform and innovation of ideological and political education of college students under the new situation of high-quality development of education. Therefore, it is of great theoretical value and practical significance to study the psychological structure and mechanism of college students in the process of Chinese cultural identity.

Clifford, American anthropologist Gertz said: our thoughts, our values, our actions, and even our emotions, like our nervous system itself, are the products of culture. College students’ identification with Chinese culture is a four-dimensional construction of “cognition emotion idea behavior” and a process of synergy. We should fully grasp the internal logical law of college students’ psychology and help college students build a positive and healthy Chinese cultural identity through a good psychological and action intervention mechanism, further forge the consciousness of the Chinese national community, and provide practical suggestions for colleges and universities to carry out ideological and political education.

#### Promote the Integration of Excellent Chinese Traditional Culture Into the Curriculum System of Colleges and Universities, and Strengthen the Cognitive Education of Traditional Culture for College Students

Colleges and universities should improve the existing curriculum system of ideological and political courses and general education, and let the excellent traditional Chinese culture enter the curriculum system of colleges and universities. First of all, we should introduce the elements and connotation spirit of the excellent Chinese traditional culture into the ideological and political classroom, so as to fully integrate the excellent Chinese traditional culture with the ideological and political education. For example, in the course of Basic Principles of Marxism, the teacher can combine the society corresponding to China’s “Datong” period with the ideal of communism; In “Ideological and Moral Cultivation and Legal Basis,” in classical classics such as the Analects of Confucius, “there must be one out of three who can be your teacher.” And other famous sentences are combined with students’ values, and the Confucian “ritual” culture is combined with the cultivation of students’ interpersonal relationships, so as to cultivate college students’ spiritual quality with Chinese excellent traditional culture.

Secondly, we should combine the regional characteristics of the school, tap the advantages of local traditional culture, and design the school-based curriculum of general education of Chinese excellent traditional culture with local characteristics. It is necessary to carry out Chinese traditional culture education to college students through general courses, public elective courses, and other forms, improve the knowledge reserve of traditional culture, strengthen college students’ cognition of Chinese traditional culture, and make college students feel the breath of traditional culture in the process of learning.

#### Expand the Aesthetic Dimension of Excellent Chinese Traditional Culture and Deepen the Emotional Identity of College Students With Traditional Culture

First of all, we should rationally understand the aesthetic elements in the roots of traditional culture and stimulate the emotional memory of the blending of explicit and implicit traditional culture. Teachers should select some artistic works, such as national dance, folk music, traditional Chinese painting, and traditional poetry, as well as some flexible cultural carriers, such as touching letters, admirable characters, and inspiring stories. Teachers should provide in-depth analysis of and convey the rich spiritual emotion and cultural aesthetics contained in it, so that college students can have emotional resonance due to good aesthetic experience when receiving education, so as to enhance the positive emotion of excellent Chinese traditional culture and promote the effect of emotional recognition of excellent Chinese traditional culture.

Secondly, we should organically introduce the emerging carrier resources of traditional culture and improve the quality and level of Chinese excellent traditional culture education. Recently, programs such as “National Treasure,” “Night at the Museum” and “Win in the Museum” have aroused the “cultural and Expo fever” of Chinese traditional culture; See China, launched in the spring of 2022, explores the famous classics in Fuzhou’s traditional culture. These new era integrated media programs use new media means and audition presentation methods to arouse people’s collective memory of Chinese traditional culture. Colleges and universities should also make effective use of these audio-visual media resources, grasp the “time, degree and effect” of traditional culture education, and enhance its attraction and appeal, so as to make Chinese traditional culture appealing, stimulate the emotional mechanism, generate a promoting effect in the process of college students’ cognition and acceptance, and promote the achievement of educational effect.

#### Highlight the Spiritual Core of Excellent Chinese Traditional Culture and Enhance College Students’ Recognition of Traditional Cultural Ideas

Colleges and universities should continue to inherit and make rational use of the genes of Chinese excellent traditional culture, make the spiritual core of traditional culture the root of contemporary college students’ cultural self-confidence, and make it the spiritual support for college students to establish socialist values. First of all, we should organically combine the elements of Chinese excellent traditional culture with modern society, and grasp the balance and internal inheritance relationship between the historicity and reality of traditional culture. For example, in the process of education, we should be good at using facts, cases, and materials to explain modern problems such as socialist market economy, democratic politics, advanced culture, social governance, and so on. We should associate the excellent Chinese traditional culture with the actual life and social situation of college students, and explain the practical difficulties faced by college students in academic, emotional, and career choices with the ideals, beliefs, humanistic spirit, and ways of life contained in the excellent Chinese traditional culture, so as to realize the application of the excellent Chinese traditional culture in this world.

Secondly, we should strengthen the management of network ideology and firmly grasp the guiding power of network public opinion. Colleges and universities should be good at using network media technology to carry out cultural self-confidence education in the context of globalization; guide college students to get rid of dross, and actively learn the achievements of excellent Chinese traditional culture. At the same time, colleges and universities should cultivate the ability of college students to identify good and bad, integrate and innovate, improve their awareness and sensitivity to the changes of the global cultural environment and multiculturalism, and enhance their ability to deal with the challenges of the conflict of global civilizations.

#### Strengthen the Practice and Cultivation of Excellent Chinese Traditional Culture and Promote the Identification of College Students’ Traditional Cultural Behavior

Colleges and universities should give full play to the main position of campus culture, constantly enrich the carrier and form of “cultivating people with culture,” open up the first classroom and the second classroom, and improve the effectiveness of “practical education.” First of all, we should build a diversified and multi-channel cultural exchange platform, and let college students show their favorite traditional cultural forms and contents through public, ceremonial, and dramatic traditional cultural performances. At the same time, in the process of cultural performance, college students continue to deepen their understanding of traditional culture, perceive and understand the emotions conveyed by traditional cultural works, so as to activate their own neural representation and enhance their emotional experience (Wang et al., 2018). We should also let the peer group obtain the collective memory of traditional culture in the emotional infusion aroused by the ritual performance, realize the meaning exchange between “behavior emotions,” and promote the generation of individual emotion with “common emotions,” so as to realize the parallel promotion of traditional cultural emotional identity and behavior identity of college students.

Then, we should extend the cultural practice space of college students, give full play to the collaborative forces of colleges and universities, enterprises, the government, and families, and create diversified traditional culture research and learning experience practice projects. Through investigation and learning, interactive sharing, investigation, and other activities, college students build their cognition of cultural relics, culture, intangible cultural heritage, cultural sites, and other traditional cultural carriers, and stimulate their learning expectations of excellent Chinese traditional culture. Through in-depth practical participation, we can deeply understand the image of traditional culture, explore the context of the Chinese nation, and improve our ability to carry forward the excellent Chinese traditions.

## Data Availability Statement

The raw data supporting the conclusions of this article will be made available by the authors, without undue reservation.

## Ethics Statement

The studies involving human participants were reviewed and approved by Fujian Normal University Ethics Committee. The patients/participants provided their written informed consent to participate in this study. Written informed consent was obtained from the individual(s) for the publication of any potentially identifiable images or data included in this article.

## Author Contributions

All authors listed have made a substantial, direct, and intellectual contribution to the work and approved it for publication.

## Conflict of Interest

The authors declare that the research was conducted in the absence of any commercial or financial relationships that could be construed as a potential conflict of interest.

## Publisher’s Note

All claims expressed in this article are solely those of the authors and do not necessarily represent those of their affiliated organizations, or those of the publisher, the editors and the reviewers. Any product that may be evaluated in this article, or claim that may be made by its manufacturer, is not guaranteed or endorsed by the publisher.
