# Supplementary material for: A Study on the Psychological Structure and Mechanism of Shared Chinese Cultural Identity of University Students
Source: Front Psychol. 2022 Jul 19;13:943989. doi: 10.3389/fpsyg.2022.943989 (PMC9343735; doi:10.3389/fpsyg.2022.943989)
Supplement: Supplementary file 1 [file Data_Sheet_1.docx]

**Questionnaire on cultural identity of College students**

PartⅠ THE BASIC INFOMATION

The first part is about the basic personal information. There is no right or wrong answer. Please tick "√" on the matching options according to your real situation.

1. What is your gender?（ ）

A.Men B.Women

1. What’s your nationality?（ ）
2. Ethnic Han B.Ethnic Minorities

3. What is your location of home?（ ）

A.Cities and towns B.Rural

4.What is your status as a student?（ ）

A.Freshman Year B.Sophomore year C.Junior year D.Senior year

E. Postgraduate student F.Doctoral student

5.What's your major?（ ）

A.literature B.Neo-confucianism C.Engineering D.Medical

E.Master of Arts F.Agriculture G.other

6.What is your political status?（ ）

A.Communist party member (including probationary member) B.League member C.The Masses

7.Do you believe in any religion?（ ）

A.No religious belief B.Religious belief

8.What's your father's education?（ ）

A.Primary school degree or above B.junior high school education

C.High school, technical secondary school or vocational middle school education D.College degree E.Bachelor degree

F. Master's degree G.Doctoral degree

9.Your father's occupation ?（ ）

A. Unskilled and semi skilled workers: such as housewives, farmers, workers, friends, pushers, fishermen, sailors, waiters, helpers, soldiers, unemployed

B Skilled workers: such as technicians, foremen, salesmen, clerks, small shopkeepers, drivers, tailors, beauticians, hairdressers, chefs, and platoon level officers

C. General public servants: such as township cadres, technicians, clerks, bankers, cashiers and general public servants

D. Professional and technical personnel and middle-level administrative personnel: such as accountants, doctors, judges, lawyers, engineers, architects, middle and senior public servants (including departments, bureaus and departments), middle school teachers, factory directors, company bosses, middle-level commercial managers

E. Senior professionals and senior administrative personnel: such as doctors with senior titles, provincial public opinion representatives, senior administrative personnel (including department and ministerial levels), teachers of colleges and universities, generals Major industrial and commercial bosses (such as chairman and general manager)

10.What's your mother's education?（ ）

A.Primary school degree or above B.junior high school education

C.High school, technical secondary school or vocational middle school education D.College degree E.Bachelor degree

F. Master's degree G.Doctoral degree

11.Your mother's occupation （ ）

A. Unskilled and semi skilled workers: such as housewives, farmers, workers, friends, pushers, fishermen, sailors, waiters, helpers, soldiers, unemployed

B Skilled workers: such as technicians, foremen, salesmen, clerks, small shopkeepers, drivers, tailors, beauticians, hairdressers, chefs, and platoon level officers

C. General public servants: such as township cadres, technicians, clerks, bankers, cashiers and general public servants

D. Professional and technical personnel and middle-level administrative personnel: such as accountants, doctors, judges, lawyers, engineers, architects, middle and senior public servants (including departments, bureaus and departments), middle school teachers, factory directors, company bosses, middle-level commercial managers

E. Senior professionals and senior administrative personnel: such as doctors with senior titles, provincial public opinion representatives, senior administrative personnel (including department and ministerial levels), teachers of colleges and universities, generals Major industrial and commercial bosses (such as chairman and general manager)

Part II

INFLUENCING FACTORS OF COLLEGE STUDENTS' IDENTIFICATION WITH CHINESE EXCELLENT TRADITION

The second part is mainly to understand the factors that may affect the identification of excellent Traditional Chinese culture in your growing environment. Please read the following views or questions carefully and tick "√" on the corresponding options according to your true feelings about whether you agree or not.

|  | Strongly Disagree | Somewhat Disagree | Not Sure | Somewhat Agree | Strongly Agree |
| --- | --- | --- | --- | --- | --- |
| 1.Your family pay attention to traditional Chinese festivals such as Spring Festival and Mid-Autumn Festival. |  |  |  |  |  |
| 2.Your family attaches great importance to traditional Chinese etiquette and customs. |  |  |  |  |  |
| 3.Your family watches TV programs about traditional Chinese culture. |  |  |  |  |  |
| 4.Your classmates like traditional Chinese culture. |  |  |  |  |  |
| 5.Your classmates enjoy western festivals. |  |  |  |  |  |
| 6.Your classmates like watching foreign movies. |  |  |  |  |  |
| 7.Your classmates pay attention to foreign media |  |  |  |  |  |
| 8.Your university offers many courses on excellent Traditional Chinese culture. |  |  |  |  |  |
| 9.Your university has held many lectures on excellent traditional Chinese culture. |  |  |  |  |  |
| 1. Your university community has held many activities on excellent traditional Chinese culture. |  |  |  |  |  |
| 11.Your professional course teachers have explained traditional Chinese culture in the professional course. |  |  |  |  |  |
| 12.Your ideological teacher explains traditional Chinese culture in the ideological and political class. |  |  |  |  |  |
| 13.Your school has organized many social activities related to traditional Chinese culture. |  |  |  |  |  |
| 14.Your city hosts many community activities related to traditional Chinese culture. |  |  |  |  |  |
| 15.There are Chinese traditional culture Exhibition halls (museums) in your city. |  |  |  |  |  |

Part III THE PSYCHOLOGICAL STRUCTURE OF COLLEGE STUDENTS' IDENTIFICATION WITH CHINESE EXCELLENT TRADITIONAL CULTURE

The third part is mainly to understand your cognition, identification, concept and behavior of traditional Chinese culture. Please read the following views or questions carefully and tick "√" on the corresponding options according to your true feelings about whether you agree or not.

1. Cognition

|  | Strongly Disagree | Somewhat Disagree | Not Sure | Somewhat Agree | Strongly Agree |
| --- | --- | --- | --- | --- | --- |
| 1. I am well aware of the contents of excellent traditional Chinese culture. |  |  |  |  |  |
| 2.I know a lot about traditional Chinese folk customs. |  |  |  |  |  |
| 3.I am very familiar with the four great Classical novels of China. |  |  |  |  |  |
| 4. I know a lot of the Chinese traditional virtue |  |  |  |  |  |
| 5. I know Chinese martial arts very well |  |  |  |  |  |
| 6.I know a lot about Traditional Chinese medicine |  |  |  |  |  |
| 7.I know a lot about China's intangible cultural heritage. |  |  |  |  |  |
| 8.I know a lot about the four great inventions of China. |  |  |  |  |  |
| 9.I know a lot about traditional Chinese opera art. |  |  |  |  |  |
| 10. I know a lot about traditional Chinese calligraphy and painting |  |  |  |  |  |
| 11.I know the Spirit of the Chinese nation well. |  |  |  |  |  |
| 12.I know a lot about traditional Han Chinese clothing. |  |  |  |  |  |
| 13.I know a lot about traditional Chinese cultural relics and place of interest . |  |  |  |  |  |
| 14.I know a lot about traditional Chinese books. |  |  |  |  |  |
| 15.I can recite many classical Chinese poems . |  |  |  |  |  |
| 16.I know a lot about Chinese traditional festivals |  |  |  |  |  |
| 17.I have a good knowledge of traditional Chinese etiquette. |  |  |  |  |  |

1. Emotion

|  | Strongly Disagree | Somewhat Disagree | Not Sure | Somewhat Agree | Strongly Agree |
| --- | --- | --- | --- | --- | --- |
| 18.I really like the excellent traditional Chinese culture. |  |  |  |  |  |
| 19. I'm willing to learn the Chinese traditional culture. |  |  |  |  |  |
| 20. I want to take traditional Chinese culture as an elective. |  |  |  |  |  |
| 21. I like learning about traditional Chinese folk customs very much. |  |  |  |  |  |
| 22. I hope the school can offer courses of excellent traditional Chinese culture. |  |  |  |  |  |
| 23. I hope that the school held more related to the Chinese excellent traditional culture of campus cultural activities. |  |  |  |  |  |
| 24.I prefer Chinese Valentine's Day to Western Valentine's Day |  |  |  |  |  |
| 25.I like watching television programs about excellent traditional Chinese culture very much |  |  |  |  |  |
| 26.I like Chinese traditional festivals better than western traditional festivals. |  |  |  |  |  |
| 27. If I see people wearing Hanfu when I hang out, I appreciate it very much. |  |  |  |  |  |
| 28. I'm proud to be a Chinese |  |  |  |  |  |
| 29.I'm very proud of learning traditional Chinese culture |  |  |  |  |  |

1. Concept

|  | Strongly Disagree | Somewhat Disagree | Not Sure | Somewhat Agree | Strongly Agree |
| --- | --- | --- | --- | --- | --- |
| 30.I think Sinology should be a compulsory course for college students. |  |  |  |  |  |
| 31.I think every Chinese should pay attention to Chinese traditional festivals. |  |  |  |  |  |
| 32.I think we should enhance college students' awareness of the excellent traditional Chinese culture. |  |  |  |  |  |
| 33.I believe that traditional Chinese culture embodies the wisdom and sweat of the Chinese people. |  |  |  |  |  |
| 34. In my opinion, traditional Chinese culture contains the humanistic spirit of generosity, kindness, modesty and prudence, and yearning for peace. |  |  |  |  |  |
| 35. I think it is meaningful to study the fine traditional Chinese culture. |  |  |  |  |  |
| 36.I think is worth inheriting Chinese excellent traditional culture |  |  |  |  |  |
| 37. I think it is the right thing for every Chinese to learn the excellent traditional Chinese culture. |  |  |  |  |  |
| 38. I think the fine traditional Chinese culture is more in line with the reality of China's development than the western culture. |  |  |  |  |  |
| 39. I agree with my family's education of excellent traditional Chinese culture. |  |  |  |  |  |
| 40. I really agree with the traditional Chinese culture education provided by the school. |  |  |  |  |  |
| 41.I think it is necessary to strengthen the education of traditional Chinese culture in ideological and political courses. |  |  |  |  |  |
| 42.I think that traditional Chinese culture is of great practical significance to the current social construction. |  |  |  |  |  |
| 43. I think we should inherit and carry forward traditional Chinese culture in innovation. |  |  |  |  |  |

1. Behavior

|  | Strongly Disagree | Somewhat Disagree | Not Sure | Somewhat Agree | Strongly Agree |
| --- | --- | --- | --- | --- | --- |
| 44. I will take the initiative to learn the excellent traditional Chinese culture. |  |  |  |  |  |
| 45. I will take the initiative to practice Chinese excellent traditional virtues. |  |  |  |  |  |
| 46. I am willing to take part in voluntary activities to protect and promote traditional Chinese culture. |  |  |  |  |  |
| 47. I am willing to read some books about traditional Chinese culture |  |  |  |  |  |
| 48. I surf the Internet for some news and information about traditional Chinese culture. |  |  |  |  |  |
| 49. I watch movies or read novels related to traditional Chinese culture. |  |  |  |  |  |
| 50.I will watch the Chinese Poetry Conference and other programs about Chinese culture. |  |  |  |  |  |
| 51. I will share with my friends what I know about traditional Chinese culture. |  |  |  |  |  |
| 52. I will discuss traditional Chinese culture with my friends in my daily life. |  |  |  |  |  |
| 53. I will learn Chinese etiquette to have a deeper understanding of traditional Chinese culture. |  |  |  |  |  |
| 54.I will halt those who slander traditional Chinese culture. |  |  |  |  |  |
